# B-Cell Lymphoma Presenting With Seventh Cranial Nerve Palsy and Mononeuritis Multiplex: A Case Report and Comprehensive Literature Review

**DOI:** 10.7759/cureus.44983

**Published:** 2023-09-10

**Authors:** Yongzhen Chen, Yilun Wang, John Corrigan, Anza B Memon

**Affiliations:** 1 School of Medicine, Saint Louis University, Saint Louis, USA; 2 School of Medicine, Texas Agricultural and Mechanical (A&M) University, Bryan, USA; 3 Department of Radiology, Henry Ford Health System, Detroit, USA; 4 School of Medicine, Wayne State University, Detroit, USA; 5 Department of Neurology, John D. Dingell Veterans Affairs Medical Center, Detroit, USA; 6 Department of Neurology, Henry Ford Health System, Detroit, USA

**Keywords:** nerve conduction studies (ncs), axonal degeneration, axonal neuropathy, lymphoma, bell’s palsy, mononeuritis multiplex

## Abstract

Diagnosing B-cell lymphoma-associated mononeuritis multiplex is challenging due to its rarity and the potential co-existence of other causes of mononeuritis multiplex. Here, we report a case of a 74-year-old male who initially presented with left cranial neuropathies followed by right-sided extremity weakness with hyporeflexia, right facial involvement, and subsequently asymmetric weakness and multifocal muscle wasting. Minor improvements were observed with multiple rounds of steroid treatment. The diffuse large B-cell lymphoma diagnosis was eventually established six months later upon a repeat mediastinal lymph node biopsy and cerebrospinal fluid cytology. A nerve biopsy demonstrated severe axonal neuropathy with loss of axons in all fascicles without evidence of vasculitis. A muscle biopsy showed atrophy in both type 1 and type 2 fibers. A presentation of mononeuritis multiplex warrants concern for B-cell lymphoma, mainly when other mechanisms of peripheral neuropathy are less likely.

## Introduction

B-cell lymphoma is the most common non-Hodgkin lymphoma (NHL). Patients may present with diverse extranodal and extramedullary symptoms [1]. One pattern of lymphoma-associated peripheral neuropathy is mononeuritis multiplex (MNM), a distinctive neuropathy type marked by painful, disproportional, and asymmetric motor and sensory symptoms involving two or more separate peripheral nerves spontaneously [2-4]. MNM is observed across a broad spectrum of conditions, including diabetes mellitus, amyloidosis, neurosarcoidosis, infections, rheumatological disorders, hematological disorders, and malignancies (lymphoma, B-cell leukemia, carcinoid tumors, and small-cell lung cancers) [3]. In lymphoma, MNM can arise from neoplastic processes, such as neurolymphomatosis (NL), where lymphoma cells directly invade the peripheral nervous system (PNS), paraneoplastic effects, or autoimmune-mediated mechanisms [2]. Diagnosing B-cell lymphoma-associated MNM can be challenging. Although its connection with malignancies is less common, MNM’s emergence should serve as an alarming indication for early workup of underlying neoplastic sources, facilitating early treatment and averting disease progression [2,5]. Here, we present a rare case of B-cell lymphoma manifesting as mononeuritis multiplex.

This study was previously presented as an abstract at the 2022 American Association of Neuromuscular and Electrodiagnostic Medicine (AANEM) annual meeting on September 23, 2022.

## Case presentation

A 74-year-old man with a past medical history of atrial fibrillation presented with an acute onset of left-sided facial weakness for two days and droopiness of the left eyelid for one day. He was discharged with oral prednisone for Bell’s palsy.

**Figure 1:**
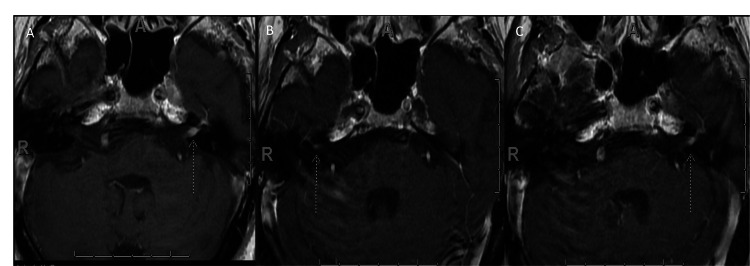
Initial brain MRI and repeat brain MRI. Initial brain MRI: T1 sagittal with gadolinium contrast shows left facial nerve enhancement (A). Repeat brain MRI: T1 sagittal with gadolinium contrast shows right facial nerve enhancement (B) and enhancement of the left internal auditory canal (C).

One month later, he developed right arm and leg weakness and an abnormal gait. Two months after his initial presentation, his left facial symptoms partially improved. Brain magnetic resonance imaging (MRI) showed asymmetric enhancement of the left facial nerve (Figure 1A). An MRI of the cervical spine showed cervical spondylosis prominently at C5-C6, with no signal changes within the cord. He displayed slightly reduced strength in the distal right upper extremity and proximal right lower extremity, diminished right patellar and Achilles reflexes, and decreased pinprick sensation in the right leg. Elevated cerebrospinal fluid protein and lambda light chains were detected (Tables 1, 2). Computed tomography (CT) of the chest showed mediastinal lymphadenopathy; however, lymph node biopsy results were benign. The patient was treated with three days of 1g pulse intravenous methylprednisolone (IVMP) and showed minor improvement.

**Table 1:** Blood test results ANA: antinuclear antibody; C-ANCA: cytoplasmic antineutrophil cytoplasmic antibodies; HIV: Human immunodeficiency virus; P-ANCA: perinuclear antineutrophil cytoplasmic antibodies, IgG: Immunoglobulin G, IgA: Immunoglobulin A, IgM: Immunoglobulin M

	Latest Reference Range & Units	Values
ANA	Negative	Negative
ANA PATTERN		Test not performed
ANA TITER 1	<1:80 Titer	Test not performed
C-ANCA	<1:20 Titer	<1:20
P-ANCA	<1:20 Titer	<1:20
Hepatitis panel		Negative
HIV		Negative
Free Light Chains Ratio	0.26 - 1.65	0.31
Kappa Light Chains	3.3 - 19.4 mg/L	21.9 (High)
Lambda Light Chains	5.7 - 26.3 mg/L	70.5 (High)
IgG, serum	700 - 1,600 mg/dL	1,111
IgA, serum	70 - 400 mg/dL	279
IgM, serum	40 - 230 mg/dL	69
Protein, Total, serum	6.58 - 8.51 g/dL	7.0
Albumin	3.73 - 5.65 g/dL	3.91
Alpha-1 Globulins	0.13 - 0.45 g/dL	0.34
Alpha-2 Globulins	0.37 - 0.93 g/dL	0.80
Beta Globulins, serum	0.69 - 1.29 g/dL	0.85
Gamma Globulins	0.58 - 1.50 g/dL	1.10

**Table 2:** CSF results ACE: Angiotensin-converting enzyme, CMV: Cytomegalovirus, CSF: Cerebrospinal fluid, HSV: Herpes simplex virus, OR: Optimal result, PCR: Polymerase chain reaction, RBC: Red blood cell, Rpt: repeat, VDRL: Venereal disease research laboratory

	Latest Reference Range & Units	Values
Tube #		3
Volume	mL	1.0
Color		Colorless
Clarity		Clear
Glucose, CSF	40 - 80 mg/dL	64
Protein, CSF	15 - 55 mg/dL	71 (High)
ACE, CSF	< OR = 15 U/L	<5
RBC	0 /cu mm	4 (High)
Total Nucleated Cell Count	0 - 5 /cu mm	3
Neutrophils, CSF	0 - 6 %	0
Basophils	0 %	0
Eosinophils	0 %	0
Lymphocytes	40 - 80 %	97 (High)
Macrophages	0 %	0
Monocytes	15 - 45 %	3 (Low)
Mononucleates	15 - 45 %	0 (Low)
VDRL, CSF		Rpt
VDRL, CSF		NONREACTIVE
West Nile IgG Abs CSF		<1.30
West Nile IgM Abs CSF		<0.90
HSV 1 and 2 DNA		Negative
CMV DNA PCR		Negative

Four months after his initial presentation, he presented with right facial droop and right upper extremity weakness. A repeat brain MRI showed left internal auditory canal enhancement and a new enhancement of the right facial nerve (Figures 1B, 1C). He received 1g of IVMP with valacyclovir, again for Bell’s palsy. A CT of the abdomen/pelvis showed small, solid-appearing cysts at the upper pole of the right kidney. Laboratory analysis ruled out monoclonal gammopathy of undetermined significance and myeloma; a paraneoplastic panel was normal. The patient was discharged on a prednisone taper.

Six months after his initial presentation, the patient had muscle wasting in the intrinsic hand muscles on the right side and in the deltoid and biceps on the left side. Physical examination showed decreased motor function in fingers and lower extremities on the right side, deltoid and biceps on the left side, and diminished reflexes in bilateral lower extremities.

Electromyography/nerve conduction studies showed asymmetric, severe sensorimotor axonal peripheral polyneuropathy with severe active and chronic denervation and motor unit drop-out, consistent with a chronic MNM pattern (Tables 3-6). The next day, he had a hypovolemic shock, worsening right upper and lower extremity weakness, and associated muscle atrophy. A nerve biopsy showed severe axonal neuropathy with 50%-60% axonal loss in all fascicles without evidence of vasculitis (Appendices). A muscle biopsy showed type 1 and type 2 fiber atrophy (Appendices). Repeat mediastinal lymph node biopsies and cerebrospinal fluid cytology showed diffuse large B-cell lymphoma (DLBCL) (Appendices). Initially, rituximab plus cyclophosphamide-doxorubicin-vincristine-prednisone (R-CHOP) was recommended for treatment. However, it was recognized that the patient was taking amiodarone for atrial fibrillation, and the combination of amiodarone and high-dose cyclophosphamide in R-CHOP may cause lung toxicity. Therefore, the patient received one treatment cycle of bendamustine and rituximab. It was not intrathecal. However, after treatment, he developed sepsis secondary to community-acquired pneumonia and died in the intensive care unit from medical complications.

**Table 3:** Sensory nerve conduction studies NR: No response

Nerve/Sites	Rec. Site	Amplitude			Latency			Distance		Temperature	
		µV			ms			mm		°C	
		Right	Left	Ref.	Right	Left	Ref.	Right	Left	Right	Left
Median - Digit II											
Wrist	Digit II	NR	6.4	≥10.0	NR	3.6	≤4.0	140	140	35.9	35.9
Ulnar - Digit V											
Wrist	Digit V	NR	9.2	≥6.0	NR	3.2	≤4.0	140	140	36.2	35.9
Radial - wrist											
Forearm	wrist	3.3	11.6	≥7.0	3.2	2.4	≤2.8	100	100	36.3	35.9
Sural - Ankle											
Calf	Ankle	NR	NR	≥4.0	NR	NR	≤4.5	140	140	34.5	34.1

**Table 4:** Motor nerve conduction studies EDB: Extensor digitorum brevis; APB: Abductor pollicis brevis; ADM: Abductor digiti minimi; FDI: first dorsal interosseous; Tib Ant: Tibialis anterior; AH: Abductor hallucis; NR: No response

Nerve/Sites	Muscle	Amplitude			Latency			Distance		Velocity			Temperature	
		mV			ms			mm		m/s			°C	
		Right	Left	Ref.	Right	Left	Ref.	Right	Left	Right	Left	Ref.	Right	Left
Median - APB														
Wrist	APB	NR	5.5	≥3.8	NR	4.0	≤4.7	80	80				36.3	36.3
Elbow	APB		5.4			7.3			220		66	≥47		36.2
Ulnar - ADM														
Wrist	ADM	0.3	5.4	≥7.9	4.3	2.8	≤3.7	80	80				36.3	36.2
B.Elbow	ADM	0.3	5.3		8.6	6.3		210	200	49	57	≥52	36.3	36.4
A.Elbow	ADM	0.3	5.2		11.6	8.4		130	120	43	58	≥43	36.3	36.3
Ulnar - FDI														
Wrist	FDI	0.2	9.9	≥9.2	4.3	3.8	≤4.0	170	180				36.2	36.1
B.Elbow	FDI	0.2	8.7		8.0	7.5		210	210	58	56	≥51	36.3	36.6
A.Elbow	FDI	0.2	7.6		11.4	9.6		130	120	38	56	≥49	36.6	36.4
Fibular - EDB														
Ankle	EDB	NR	0.4	≥1.1	NR	3.8	≤6.5	80	80				34.9	34.5
Pop fossa	EDB		0.3			11.9			380		47	≥38		34.5
Fibular - Tib Ant														
Fib Head	Tib Ant	NR	4.8	≥1.5	NR	3.6	≤4.9		100			≥43	34.7	34.5
Pop fossa	Tib Ant		4.8			5.9			100		45	≥43		34.5
Tibial - AH														
Ankle	AH	2.6	0.8	≥1.1	3.9	5.5	≤6.1	80	80				34.8	34.7
Pop fossa	AH	1.7			14.4			400		38		≥39	34.8	

**Table 5:** F wave latency EDB: Extensor digitorum brevis; APB: Abductor pollicis brevis; ADM: Abductor digiti minimi; AH: Abductor hallucis; F Lat: F wave latency; Temp: Temperature; NR: No response

Nerve	F Lat		
	ms		
	Right	Left	Reference
Ulnar - ADM	NR	27.8	≤31.5
Tibial - AH	54.1	NR	≤61.4
Fibular - EDB		NR	≤61.2
Median - APB		27.9	≤31.6

**Table 6:** Needle electromyography MUAP: Motor unit action potential; IA: Insertional activity; PSW: Positive sharp waves; Fib: Fibrillation; Fasc: Fasciculation; Amp: Amplitude; Dur: Duration; Polys: Polyphasia; N: Normal

EMG Summary Table										
	Spontaneous					MUAP				
Muscle	IA	PSW	Fib	Fasc	Other	Effort	Recruitment	Amp	Dur.	Polys
R. Deltoid	N	N	N	N	N	N	N	N	N	N
R. Biceps brachii	N	N	N	N	N	N	N	N	N	N
R. Triceps brachii	N	N	N	N	N	N	N	N	N	N
R. Flexor carpi radialis	N	N	N	N	N	N	-2	N	N	+2
R. Flexor digitorum profundus (Ulnar)	Incr	Sustained	N	N	N	N	-3	+2	+2	+3
R. Extensor indicis proprius	N	N	N	N	N	N	N	N	N	N
R. First dorsal interosseous	Incr	Sustained	4+	N	N	N	-4			
R. Abductor pollicis brevis	Incr	Sustained	3+	N	N	N	-4			
R. Vastus lateralis	Incr	Sustained	2+	N	N	N	-2	+1	+1	+2
R. Tibialis anterior	Incr	Sustained	3+	N	N	N	-4			
R. Peroneus longus	Incr	Sustained	4+	N	N	N	-4			
R. Medial Gastrocnemius	Incr	Sustained	3+	N	N	N	-2	N	N	+2
R. Extensor digitorum brevis	Incr	Sustained	3+	N	N	N	-4			
L. Deltoid	Incr	Sustained	2+	N	N	N	-3	+1	+2	+3
L. Biceps brachii	Incr	Sustained	2+	N	N	N	-3	+2	+1	+1
L. Triceps brachii	N	N	N	N	N	N	N	N	N	N
L. Flexor carpi radialis	N	N	N	N	N	N	N	N	N	N
L. Extensor indicis proprius	N	N	N	N	N	N	N	N	N	N
L. First dorsal interosseous	N	N	N	N	N	N	N	N	N	N
L. Abductor pollicis brevis	N	N	N	N	N	N	N	N	N	N

## Discussion

B-cell lymphoma is a type of lymphoma that originates in the B-cells [6]. It constitutes approximately 85% of the NHL [7]. Common subtypes of B-cell lymphoma include large B-cell lymphoma, follicular lymphoma, marginal zone B-cell lymphoma, Burkitt lymphoma, and mantle cell lymphoma [6]. Neurological complications associated with lymphoma are more prevalent in NHL, particularly B-cell NHL [8]. NHL tends to infiltrate nerves diffusely, while Hodgkin lymphoma (HL) is more likely to cause PNS immunological disorders, such as inflammatory plexopathy or Guillain-Barré syndrome [8]. Higher lymphoma grades increase the likelihood of nervous system involvement [8]. PNS involvement occurs in 5% of lymphoma cases [4]. In NHL, PNS manifestations include radiculopathies and plexopathies (often due to paraneoplastic syndrome or direct nerve infiltration), focal neuropathies (resulting from systemic lymphoma spread), and peripheral neuropathy (mainly due to chemotherapy) [9]. High-grade lymphomas, such as Burkitt and intravascular lymphoma, can involve the central nervous system (CNS) [9,10]. Vallat et al. categorized peripheral neuropathies unrelated to chemotherapy in NHL into four groups based on mechanisms [11]:

Group I: Direct invasion of neoplastic cells into a peripheral nerve, e.g., NL, as supported by nerve biopsy;

Group II: NHL causes demyelinating neuropathies with monoclonal immunoglobulin (IgM) with anti-myelin activity, as supported by serum electrophoresis;

Group III: Autoimmune-mediated peripheral neuropathies such as Guillain-Barré syndrome or chronic inflammatory demyelinating polyneuropathy (CIDP), as supported by an autoimmune profile;

Group IV: Undermined, possibly paraneoplastic etiologies associated with the tumor.

MNM is a distinctive form of painful sensorimotor peripheral neuropathy characterized by the asymmetric and asynchronous involvement of two or more distinct nerves in random anatomical regions of the body [2,3]. In our case, MNM presented as early asymmetric cranial neuropathies followed by subacute-to-chronic progression of asymmetrical multifocal extremity weakness with hyporeflexia. MNM can be linked to various conditions, including vasculitis, infections, diabetes, autoimmune diseases, malignancies, or paraneoplastic syndromes [3].

A study found that when it comes to lymphoma-associated peripheral neuropathy, two patterns are commonly observed: symmetrical polyneuropathy, or MNM [4]. In this study, most NL manifests MNM, while paraneoplastic conditions like CIDP, sensory ganglionopathy, and vasculitic neuropathy lean towards a symmetrical polyneuropathy pattern [4]. When there is no clear evidence of NL or paraneoplastic neuropathy, MNM is more likely in B-cell lymphoma, whereas T-cell lymphoma is more likely to cause symmetrical polyneuropathy [4]. Therefore, B-cell lymphoma-associated MNM is commonly secondary to either NL or undetermined mechanisms. In our case, a nerve biopsy did not show a direct lymphoma invasion, which makes NL less likely, and the absence of abnormal IgM points away from Group II. The best fit based on Vallat's classification system is Group IV.

Most NL is associated with B-cell NHL, particularly DLBCL [4,12-14]. NL invades nerves through the perineural spread, which is distinct from leptomeningeal disease-the spread of malignancy to cerebrospinal fluid involving leptomeninges encompassing the arachnoid, subarachnoid space, and pia mater [15]. The mechanism of NL is most likely demyelination unrelated to macrophages at the site of lymphomatous cell invasion and axonal degeneration distal from the location of the lymphomatous cell invasion, distinguishing it from paraneoplastic peripheral neuropathies [4]. NL can emerge at any stage of lymphoma, with 10% occurring initially and 25% concurrently, while most cases arise during disease recurrence or following multiple treatment cycles [12,13,16]. The preferred sites of NL include the lumbosacral plexus, brachial plexus, sciatic nerve, femoral nerve, and trigeminal nerve [12,13]. There have been occasional cases of ocular nerve involvement reported [17]. Non-NL B-cell lymphoma-associated MNM is extremely rare, especially as an isolated manifestation [2,18].

Diagnosing B-cell lymphoma-associated MNM relies on clinical presentation, pathologic, and radiologic findings. In the case of NL, MRI shows thickening and enhancement of involved cranial nerves, nerve roots, plexus, and trunks of peripheral nerves, particularly post-gadolinium administration. However, the sensitivity of MRI is often limited due to the patchy distribution or small size of lesions [16,19]. Similar MRI findings can also be seen in inflammatory neuropathies [14]. Recently, positron emission tomography/computed tomography (PET/CT) with 2-deoxy-2-18F-fluoro-D-glucose (18F-FDG) has been heavily utilized for diagnosing NL due to its ability to locate involved peripheral nerves. The strong FDG uptake in affected structures, indicative of hypermetabolism, aids in identifying potential sites for nerve biopsy [14,16]. The reported sensitivity of 18F-FDG-PET/CT for NL ranges from 87.5% to 100% [16]. A nerve biopsy remains the gold standard, with a diagnostic yield of 84% [16]. Sural and peroneal nerves are commonly chosen biopsy sites [16]. However, a biopsy can have false-negative results due to patchy involvement. In addition, the utilization of biopsy is limited due to invasiveness and concern about possible postoperative neurological deficits [13,16]. Conversely, fluid cytopathologic evaluation exhibits a low diagnostic yield in NL, ranging from 20% to 40% [13,14,19,20]. In non-NL B-cell lymphoma-associated MNM, nerve biopsy will not reveal direct lymphoma invasion, whereas lymph node biopsy may still indicate lymphoma presence, as evidenced in our case.

Treatment of NL follows a similar approach to primary CNS lymphoma treatment, often starting with staging with vitreous, contrast-enhanced MRI images of the whole neuraxis and whole-body FDG-PET [14,19]. Subsequently, most patients receive systemic chemotherapy alone or in combination with intrathecal chemotherapy or external beam radiotherapy [19]. Chemotherapy alone yields a response rate of 82% [20]. For patients with isolated nervous system involvement, intravenous methotrexate is the first-line treatment, with clinical improvement typically observed after six cycles of therapy [19]. Adding rituximab to chemotherapy may improve the survival of patients with DLBCL, but its impact on NL is limited [20]. Intrathecal chemotherapy may treat the leptomeningeal involvement caused by lymphoma but can cause insufficient nerve infiltration [19]. One study reported a relapse-free period for one year with salvage ESHAP therapy (etoposide, methylprednisolone, cytosine arabinoside, and cisplatin) followed by BEAM (BCNU, etoposide, cytarabine, and melphalan) chemotherapy with stem-cell transplant [20]. In a case involving non-NL MNM due to intravascular large B-cell lymphoma, the patient received chemotherapy consisting of doxorubicin, rituximab, cyclophosphamide, and vincristine for approximately six months, along with daily oral prednisolone. Despite the inferior prognosis of intravascular lymphoma, this patient had a favorable survival of more than two years following the initial presentation [5]. Another patient with non-NL MNM experienced symptom resolution after plasmapheresis [2]. The prognosis tends to be unfavorable once NL develops. In a case series, all patients died four months after the diagnosis of NL [20]. The median overall survival of NL is 10 months from the initial diagnosis [19].

Due to the complicated nature of MNM diagnosis, nearly half of the reported NL did not establish a clinical or histopathologic diagnosis until autopsy [19]. Connecting the clinical presentations of MNM to an underlying B-cell lymphoma can be challenging due to several reasons:

1. MNM can emerge in a broad spectrum of diseases [3].

2. B-cell lymphoma-associated peripheral neuropathy, particularly B-cell lymphoma-associated MNM, is rare [2,5]. A study showed that the relative incidence of NL was only about 3% in patients with newly diagnosed intermediate- or high-grade NHL annually [19].

3. The neuro-injuries observed in B-cell lymphoma-associated MNM can be complicated by the potential co-existence of paraneoplastic disorders, inflammation, infection, or PNS involvement secondary to chemotherapy and radiology [12,14].

4. Peripheral neuropathy may precede systemic symptoms or lymphoma detection, or patients with B-cell lymphoma may present with only MNM without typical lymphoma symptoms, as seen in our case [4,5,13].

5. The diagnosis of NL requires a nerve biopsy, which could be limited by procedural accessibility and concerns about a potentially more significant deficit caused by the invasiveness [14]. Negative cerebrospinal fluid and bone marrow analyses might divert physicians from suspecting an underlying lymphoma [12].

## Conclusions

This B-cell lymphoma-associated MNM case report aims to underscore the significance of considering lymphoma as a potential cause of asymmetric cranial neuropathies and asymmetric, asynchronous, painful, sensorimotor axonal peripheral polyneuropathy. Although B-cell lymphoma-associated MNM is uncommon and its clinical presentation can mimic other commonly seen peripheral neuropathies, its prognosis and response to treatment are unique. Timely workup of lymphoma is warranted when empiric treatment of neuropathies yields no improvement. Future investigations into B-cell lymphoma-associated MNM can help elucidate the mechanisms, diagnostic approaches, and therapeutic strategies for this rare condition.

## Appendices

Operation/Specimen

A: Gastrocnemius Muscle for Light Microscopy B: Gastrocnemius Muscle for Histochemistry C: Gastrocnemius Muscle for Electron Microscopy D: Nerve, left sural, biopsy E: Nerve, left sural, biopsy for Immunofluorescence F: Nerve, left sural, biopsy for Electron Microscopy G: Lymph node, mediastinal, cytology H: Lymph node, mediastinal, cytology I: Lymph node, mediastinal, lymphoma protocol, biopsy J: Cerebrospinal fluid, cytology

Diagnosis

A, B, C. Skeletal muscle, gastrocnemius, biopsy: Severe atrophy. See Comment and Microscopic description. D, E, F. Peripheral nerve, left sural, biopsy:  Severe axonal neuropathy. See Comment and Microscopic description. G, H. Lymph node, mediastinal, cytology: Atypical lymphoid proliferation, suspicious for B-cell non-Hodgkin lymphoma. See Microscopic description. I.  Lymph node, mediastinal, biopsy: High-grade B-cell lymphoma with morphologic features of diffuse large B-cell lymphoma (DLBCL). See comment. J. Cerebrospinal fluid: Suspicious for malignant lymphoma. See comment.

Comment

A, B, C. Muscle Biopsy

This muscle shows severe atrophy with fatty infiltration. Both type 1 and Type 2 fibers are involved. The atrophic fibers are rounded rather than angulated. While both fiber types are involved, other features of long-standing neurogenic atrophy, such as fiber type grouping, group atrophy, and target fibers, are not present. There is no inflammation, muscle necrosis/regeneration, or upregulation of MHC-1. No perifascicular atrophy is seen. Please correlate with clinical, laboratory, and electrophysiologic findings.

D, E. F. Nerve Biopsy

The biopsy shows severe axonal neuropathy with 50%-60% axonal loss in all fascicles. The axonal loss involves the myelinated axons, large and small. There is no significant active axonal degeneration. There is no evidence of significant axonal regeneration. There is no variation in the degree of axonal loss within or among the fascicles. No endoneurial or epineurial inflammation is seen, and there is no vasculitis. The etiology of the neuropathy cannot be determined from the histologic examination, as is the case in the majority of axonal neuropathies. In the context of the recently diagnosed lymphoma in this patient, a paraneoplastic etiology of the neuromuscular pathology should be considered. Please correlate with clinical, laboratory, and electrophysiologic findings.

I.  Lymph Node, Mediastinal, Biopsy

The majority of the tissue shows evidence of high-grade B-cell lymphoma, which shows no aberrant expression of CD5. Additionally, a few minute portions of the tissue are comprised of B-lymphocytes with aberrant expression of CD5. Along with the flow cytometry findings (CD5-positive/CD23-positive B-cell clone), these areas are suspicious (but not diagnostic) of DLBCL transformation from a low-grade B-cell lymphoma; however, definitive evidence of this possibility cannot be established due to the scarcity of tissue.

The high-grade B-cell lymphoma shows the following characteristics:

- Non-germinal center subtype based on the Hans algorithm (CD10 negative, BCL6 positive, and MUM1 positive) - BCL2 is positive, and c-MYC is positive. Based on this immunoprofile, this high-grade B-cell lymphoma would be considered a double expressor - MIB1 proliferation index is >95% - CD30 immunostain is negative - EBER-ish is negative for EBV

Flow Cytometry: The bright CD45+ cells with small-to-large lymphoid light scatter are analyzed by flow cytometry. These are a mixture of mature T- and monoclonal B-lymphocytes. The latter show the following composite antigen profile: Positive: CD19, CD20, CD5, CD23, immunoglobulin lambda light chain (dim). Negative: CD3, CD10, CD11c, CD38 and immunoglobulin kappa light chain.

The above antigen profile is consistent with a CD5+/CD23+ B-cell lymphoproliferative disorder. Please correlate with routine histology for a final diagnosis.

J. Cerebrospinal Fluid

Flow Cytometry: The bright CD45+ cells with small-to-large lymphoid light scatter are analyzed by flow cytometry. Lymphoid cells account for approximately 27% of total analyzed events in the sample, and they are predominantly (approximately 71% of lymphs) B-cells. These have a composite antigen profile of CD19+ (dim), CD5 Neg., CD23 Neg., with a heterogeneous but restricted lambda light chain pattern. Flow cytometry is positive for the clonal population of B-cells. Please correlate with cytology as well as other relevant clinical and laboratory data.

Microscopic description

A, B, C. Muscle Biopsy

H&E, enzyme histochemistry, and special stains: H&E, Congo red, and all immunostains except (MHC-1) are performed on separate slides from block A1 (fixed paraffin embedded tissue). All other histochemical stains and MHC-1 immunostaining were performed on separate slides from block B1 (frozen tissue).

H and E-stained paraffin, as well as cryostat sections, show a severely atrophic muscle with fatty infiltration. The atrophic fibers are round, with scattered hypertrophic myofibers in between. No perifascicular atrophy is seen. There is no significant perimysial, endomysial, or epimysial inflammation. No vasculitis is seen. No necrotic or regenerating myofibers are seen. However, scattered hypercontracted and degenerating myofibers are noted. Other myopathic features (interstitial fibrosis or increased internal nuclei) are absent. CD163 immunostain highlights macrophages in the interstitium as well as the perimysium. CD3 immunostain shows a few scattered immunoreactive cells. The MHC-1 immunostain shows no upregulation and highlights the endomysial capillaries.

Modified trichrome-stained sections demonstrate a normal punctate sarcoplasmic staining pattern without evidence of ragged red fibers, rimmed vacuoles, or other abnormal sarcoplasmic accumulations. ATPase preparations at pH 4.3, 4.6, and 9.5 show a slight predominance of type I myofibers. No definite fiber type grouping or group atrophy is noted. NADH-stained preparations reveal scattered myofibers with disruption of intermyofibrillar network. No target fibers are seen. Esterase-stained preparation highlights the occasional motor end plate. PAS-stained preparations reveal no increase in glycogen stores. There is no evidence of an increase in neutral lipid stores in oil red O preparations. Alkaline phosphatase activity is not increased. Myophosphorylase and adenylate deaminase stains confirm the presence of these enzymes in the muscle fibers. No COX-negative fibers with abnormal mitochondria (SDH/PMS, COX/SDH) are noted. Congo red is negative for amyloid deposition.

Thin Paragon-stained sections obtained from five Epon-embedded blocks were examined with a light microscope. There is no evidence of abnormal structures or deposits. The sections show similar findings to the paraffin-embedded and frozen tissue described above.

D, E, F. Nerve Biopsy

H&E and special stains: An H&E stain and special stains/ immunostains were performed on block D1 (formalin-fixed tissue) with adequate controls. H&E sections contain 17 fascicles of peripheral nerve. A few scattered myelin ovoids and digestion chambers were seen. The endoneurial and perineurial microvasculatures are unremarkable. There was no evidence of vasculitis. Special stains: There are no amyloid deposits identified on the Congo red stain. Trichrome stain and Luxol fast blue stains highlight patchy loss of myelin staining. No iron deposition is seen.

Immunohistochemistry: Neurofilament stain highlights axonal architecture with patchy loss of axonal density. No giant axons are seen. CD 163 immunostain highlights scattered perineural and endoneural macrophages. CD3 immunostain shows a few scattered perineural and endoneural T cells. B cells (CD20-positive) are virtually absent.

Plastic embedded material: Thin Paragon-stained sections obtained from two Epon-embedded blocks were examined with a light microscope. A total of 13 nerve fascicles are seen with severe loss large and small myelinated axons. The loss is uniform across the fascicles. No myelin ovoids or thinly myelinated axons are noted.  There are no regenerating clusters or onion bulbs seen.  No giant axons, myelin abnormalities, or abnormal deposits are identified. The endoneurial and perineurial microvasculatures are unremarkable. There is no inflammatory infiltrate and no vasculitis.

G, H. Lymph Node Cytology

Slides and cell blocks were reviewed and showed atypical CD45+ lymphoid proliferation of mainly moderate-size CD20+ and PAX-5+ B cells with cytological atypia associated with only a few scattered CD3+ T cells. AE1/AE3 (epithelial marker) and synaptophysin are negative.
 

## References

[1] Liu Y, Barta SK (2019). Diffuse large B-cell lymphoma: 2019 update on diagnosis, risk stratification, and treatment. Am J Hematol.

[2] Sheikh AA, Sheikh AB, Tariq U, Siddiqui FS, Malik WT, Rajput HM, Ahmad I (2018). Paraneoplastic mononeuritis multiplex: a unique presentation of non-Hodgkin lymphoma. Cureus.

[3] Sutaria RB (2018). Mononeuritis multiplex. Musculoskeletal Sports and Spine Disorders: A Comprehensive Guide.

[4] Tomita M, Koike H, Kawagashira Y (2013). Clinicopathological features of neuropathy associated with lymphoma. Brain.

[5] Haqi-Ashtiani B, Moghaddam P, Barzkar F, Zare Mehrjerdi A, Almasi-Dooghaee M (2023). Mononeuropathy multiplex as an uncommon presentation of intravascular lymphoma: A case report. Clin Case Rep.

[6] Alaggio R, Amador C, Anagnostopoulos I (2022). The 5th edition of the World Health Organization classification of haematolymphoid tumours: lymphoid neoplasms. Leukemia.

[7] Dotan E, Aggarwal C, Smith MR (2010). Impact of rituximab (rituxan) on the treatment of B-cell non-Hodgkin’s lymphoma. P T.

[8] Kelly JJ, Karcher DS (2005). Lymphoma and peripheral neuropathy: a clinical review. Muscle Nerve.

[9] Giglio P, Gilbert MR (2006). Neurologic complications of non-Hodgkin's lymphoma. Curr Hematol Malig Rep.

[10] Grisold W, Grisold A, Marosi C, Meng S, Briani C (2015). Neuropathies associated with lymphoma(†). Neurooncol Pract.

[11] Vallat JM, De Mascarel HA, Bordessoule D, Jauberteau MO, Tabaraud F, Gelot A, Vallat AV (1995). Non-Hodgkin malignant lymphomas and peripheral neuropathies--13 cases. Brain.

[12] DeVries AH, Howe BM, Spinner RJ, Broski SM (2019). B-cell peripheral neurolymphomatosis: MRI and (18)F-FDG PET/CT imaging characteristics. Skeletal Radiol.

[13] Gupta M, Pasricha S, Ahmed R, Choudhury PS (2023). A case series of neurolymphomatosis: role of fluorodeoxyglucose positron emission tomography-computed tomography scan reiterated. Indian J Nucl Med.

[14] Shree R, Goyal MK, Modi M (2016). The diagnostic dilemma of neurolymphomatosis. J Clin Neurol.

[15] Fritzhand SJ, Esmaeli B, Sun J, Debnam JM (2021). Primary disease sites and patterns of spread in cases of neurolymphomatosis in the orbit associated with lymphoma. Cancer Imaging.

[16] Fatima N, Zaman MU, Zaman A, Zaman SU (2020). Neurolymphomatosis - Rare presentation in non-Hodgkin's lymphoma: The role of (18)F-fluorodeoxyglucose positron-emission tomography and computerized tomography imaging. World J Nucl Med.

[17] Liu KC, Hennessey MA, McCall CM, Proia AD (2018). Ocular involvement in neurolymphomatosis. Am J Ophthalmol Case Rep.

[18] Lynch KM, Katz JD, Weinberg DH, Lin DI, Folkerth RD (2012). Isolated mononeuropathy multiplex--a rare manifestation of intravascular large B-cell lymphoma. J Clin Neuromuscul Dis.

[19] Baehring JM, Damek D, Martin EC, Betensky RA, Hochberg FH (2003). Neurolymphomatosis. Neuro Oncol.

[20] Gan HK, Azad A, Cher L, Mitchell PL (2010). Neurolymphomatosis: diagnosis, management, and outcomes in patients treated with rituximab. Neuro Oncol.

